# Sooty Mold Detection on Citrus Tree Canopy Using Deep Learning Algorithms

**DOI:** 10.3390/s23208519

**Published:** 2023-10-17

**Authors:** Bryan Vivas Apacionado, Tofael Ahamed

**Affiliations:** 1 Graduate School of Science and Technology, University of Tsukuba, 1-1-1 Tennodai, Tsukuba 305-8577, Japan; bvapacionado@up.edu.ph; 2 Institute of Life and Environmental Sciences, University of Tsukuba, 1-1-1 Tennodai, Tsukuba 305-8577, Japan

**Keywords:** sooty mold, citrus, deep learning, YOLO, CenterNet

## Abstract

Sooty mold is a common disease found in citrus plants and is characterized by black fungi growth on fruits, leaves, and branches. This mold reduces the plant’s ability to carry out photosynthesis. In small leaves, it is very difficult to detect sooty mold at the early stages. Deep learning-based image recognition techniques have the potential to identify and diagnose pest damage and diseases such as sooty mold. Recent studies used advanced and expensive hyperspectral or multispectral cameras attached to UAVs to examine the canopy of the plants and mid-range cameras to capture close-up infected leaf images. To bridge the gap on capturing canopy level images using affordable camera sensors, this study used a low-cost home surveillance camera to monitor and detect sooty mold infection on citrus canopy combined with deep learning algorithms. To overcome the challenges posed by varying light conditions, the main reason for using specialized cameras, images were collected at night, utilizing the camera’s built-in night vision feature. A total of 4200 sliced night-captured images were used for training, 200 for validation, and 100 for testing, employed on the YOLOv5m, YOLOv7, and CenterNet models for comparison. The results showed that YOLOv7 was the most accurate in detecting sooty molds at night, with 74.4% mAP compared to YOLOv5m (72%) and CenterNet (70.3%). The models were also tested using preprocessed (unsliced) night images and day-captured sliced and unsliced images. The testing on preprocessed (unsliced) night images demonstrated the same trend as the training results, with YOLOv7 performing best compared to YOLOv5m and CenterNet. In contrast, testing on the day-captured images had underwhelming outcomes for both sliced and unsliced images. In general, YOLOv7 performed best in detecting sooty mold infections at night on citrus canopy and showed promising potential in real-time orchard disease monitoring and detection. Moreover, this study demonstrated that utilizing a cost-effective surveillance camera and deep learning algorithms can accurately detect sooty molds at night, enabling growers to effectively monitor and identify occurrences of the disease at the canopy level.

## 1. Introduction

**Figure 1:**
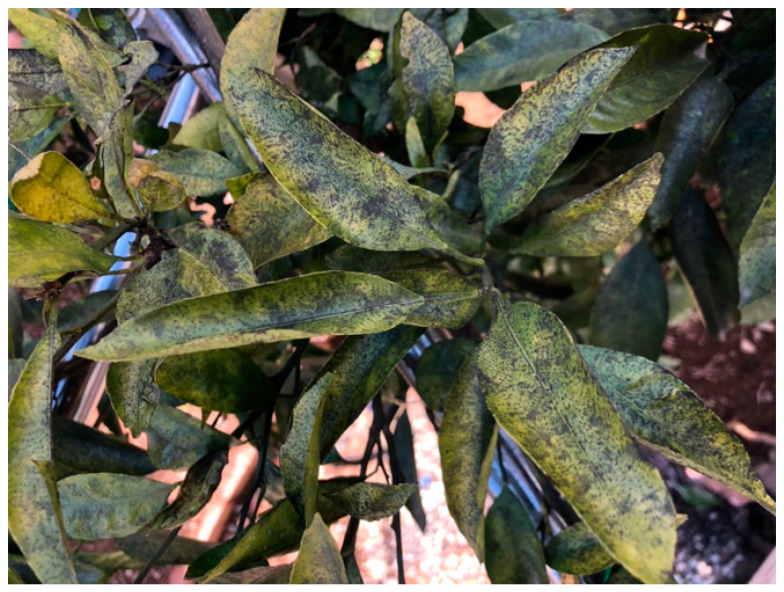
Sooty mold infection build-up on the surface of citrus leaves.

Crop pests are one of the major problems in crop production globally, reducing crop yield in quality and quantity. According to the Food and Agriculture Organization (FAO) report in 2019, between 20 and 40% of production is lost annually due to pest damage, and crop disease infection contributes to approximately USD 220 billion loss per year [1]. This is reflected in the production of citrus, one of the popular major sources of vitamin C and regarded as one of the most important and highly cultivated fruit families in the world [2,3]. Citrus diseases such as huanglongbing (HLB), leprosis, and citrus canker pose a potential yield loss of up to 80% [4]. Moreover, fungi are frequent and rampant in citrus production. Sooty mold is one of the most common occurrences, which is caused by fungi that develop and feed on honeydew produced by sap-sucking insects such as whiteflies, scale insects, aphids, psyllids, and mealy bugs [5]. The fungi create an unpleasant black surface build-up of mycelia on the stem, fruits, and leaves affecting the plants’ photosynthetic ability (Figure 1) [6]. In a study conducted by Insausti and his colleagues (2015) on orange leaves, they found that sooty mold can reduce the photosynthetic photon flux density (PPFD) by 44 to 74%, lowering photosynthesis [7]. In addition, copious sooty mold incidence may cause a reduction in fruit quality and delay fruit color development [5]. Thus, monitoring and detecting the presence of sooty mold on citrus is important to combat its negative effect on photosynthesis, fruit quality, and yield. However, most orchards still rely on manual disease monitoring, which is labor-intensive and sometimes inaccurate. Conventional disease diagnosis requires experience and expert visual assessment on-site to identify disease occurrences that can be expensive and inconvenient [8]. To address these challenges, many recent studies have focused on automating the detection and identification of plant diseases through the help of computer vision and Internet of Things (IoT) technologies. The proliferation of devices with cameras and accessible internet, coupled with the advancement in computer technology, rendered machine learning and image processing techniques, made it possible for automated detection and recognition of crop diseases [9,10].

In recent years, image recognition based on deep learning (DL) has been significantly important in detecting disease and pest damage on plants [11]. However, one of the main challenges of researchers working in this field is the acquisition of suitable and sizeable amount of image datasets for the DL models to perform reliable disease detection. Researchers worldwide have developed open-access datasets of different plant diseases that can be used by other researchers. For instance, Parez et al. (2023) explored plant disease detection using two popular publicly available datasets from PlantVillage (PV) and Data Repository of Leaf Images (DRLI) to produce a new dataset called Plant Composite [12]. The three datasets were used in GreenVit, a fine-tuned version of vision transformers (ViT) to classify healthy and unhealthy conditions of various plants with 99% accuracy. However, not all publicly available datasets are suitable or sufficient for some studies. Khan et al. (2022) collected their own images and created a new dataset for wheat disease classification of brown- and yellow-rusted disease [13]. Image data collection and disease detection can also be expensive and complicated, as most of the recent studies used high-end thermal and multispectral cameras with complex algorithms to detect crop diseases. A study conducted by Yang et al. (2019) used a Fluke TiS20 infrared thermal camera and multiple steps to detect disease on tea plants, including the conversion of RGB images to HSV, splitting color components, thresholding, color identification, converting images to gray, and noise removal [14].

To provide solutions to the challenges and limitations of the aforementioned works, this study aims for a simple, efficient, and cost-effective alternative for disease monitoring, detection, and identification using DL algorithms. The primary achievements of our research include the following:

- The study used an affordable home surveillance camera with night vison capability to capture sooty mold infections on citrus canopy at night in a field environment, thereby minimizing the effects of varying light conditions during the day to divert on using expensive cameras;
- All images were collected remotely using IoT technology to eliminate the need for an on-site data collection;
- The study provided a simple alternative on sooty mold disease detection by using less image preprocessing and easy, light, one-stage detection models: YOLOv5m, YOLOv7, and CenterNet;
- A comparative analysis of three DL algorithms has been conducted to detect sooty mold infection on the citrus canopy. The highest detection accuracy was achieved at 74.4% using YOLOv7, which can be utilized in real-time orchard disease monitoring and detection.

The remainder of the article is structured into multiple sections. Section 1 discusses the effects of sooty mold infection on citrus plants specifically, and the traditional and recent developments in the monitoring, detection, and identification of crop diseases. Section 2 enumerates related research studies on detecting and identifying diseases using different convolutional neural networks (CNNs) for different crops. Section 3 presents the methodology, including the data collection and training, to detect sooty mold infection on citrus. Section 4 and Section 5 report the results of applying DL algorithms to sooty mold detection on citrus canopy. Finally, Section 6 summarizes this paper by defining conclusions and future plans for the study.

## 2. Related Works

In recent years, DL has achieved significant advancements in crop disease monitoring and detection, significantly surpassing conventional techniques [15]. Currently, early detection of diseases through DL plays a significant role in agricultural production and decision-making by employing DL algorithms in plant disease recognition that can minimize the drawbacks associated with conventional manual selection of disease, leading to more object extraction from plant disease characteristics [16].

Single-stage detectors such as YOLO and CenterNet are reported to detect crop damage and disease quickly, as they require fewer computations and perform faster by viewing object detection in a straightforward regression task, analyzing input images, and learning to predict both the probabilities of different classes and the coordinates of bounding boxes [17]. A study conducted by Uğuz et al. (2023) in citrus fruits to detect *Alternaria alternata* and thrips diseases succeeded in obtaining 99% AP using YOLOv5 [18], while Soeb et al. (2023) demonstrated better performance of YOLOv7 in detecting tea leaf diseases in natural environments using a Canon EOS 80D Single-Lens Reflex (SLR) camera compared to previous detection models such as CNN, deep CNN, DNN, and AX-Retina [19]. They achieved 97.3% detection accuracy with 96.7% precision, 96.4% recall and 98.2% mAP. Dananjayan et al. (2022) developed a new dataset of infected citrus leaves with precise annotations and multiple classes called CCL’20 on single leaf images and found that CenterNet predicts citrus leaf diseases at an early stage with high accuracy compared to other object detection models [20].

In contrast, Syed Ab Rahman et al. (2022) employed a two-step deep CNN based on Faster R-CNN to detect citrus diseases [21]. The model delivered a 94.37% accuracy in detecting citrus black spot, citrus bacterial canker, and Huanglongbing. However, the process involved numerous data preprocessing to prepare the images for training. All images were first converted to grayscale using histogram equalization to produce images with the same intensity range. Then, the gray images were transformed into binary images using a thresholding function.

In addition to the advancement in DL algorithms, several studies have utilized unmanned aerial vehicle (UAV) technologies to monitor and detect crop diseases at the canopy level. Shahi et al. reviewed numerous works on crop disease detection, highlighting machine learning and DL techniques using UAV-based remote sensing [22]. Abdulridha and colleagues also used a UAV-based remote sensing technique to detect citrus canker using a Resonon Pika L 2.4 hyperspectral camera mounted on a UAV (DJI Matrice 600, Pro Hexacopter) and achieved 100% classification accuracy for detecting citrus canker on tree canopies in orchards [23]. Meanwhile, DadrasJavan et al. (2019) detected citrus greening on low-altitude multispectral images using a Micasense RedEdge multispectral camera attached to an unmanned aerial vehicle [24]. They obtained an 81.75% classification result that validated the potential of low-altitude multispectral imagery in fast citrus greening orchard detection.

However, without the use of UAVs and specialized cameras, some of the studies had limitations on capturing canopy level images in field conditions. Most mid-range cameras including smartphones can only capture close-up images of infected individual leaves in field environment and struggle to produce reliable field canopy images due to varying light conditions. In consequence, studies on sooty mold detection frequently operate on complex algorithms and close-up images on the field environment to avoid using expensive cameras and or images captured indoors. For instance, a study conducted by Khanramaki et al. (2021) proposed an ensemble of DL algorithms to detect citrus pests such as leafminer, pulvinaria, and sooty mold using close-up leaf images captured on a natural environment by various smartphone models [25]. The ensemble involved three levels of diversity including a classifier level, feature level, and data level. At the classifier level, four CNN architectures were used (AlexNet, VGG16, ResNet 50, Inception-ResNet-v2). At the feature level, the original RGB images were transformed into several spaces such as wavelet, Principle Component Analysis (PCA), and Lab color space. In addition, the data level used a bootstrap strategy. The authors achieved the highest accuracy of 99.04% using their proposed ensemble. The complex process of image transformation on images captured in field conditions was necessary to minimize the effect of fluctuating light conditions. According to Albattah et al. (2022), accurate identification and classification of plant diseases in the outside environment pose significant challenges primarily due to varying field conditions [8]. The presence of low-intensity information in both the image background and foreground, substantial color resemblance between healthy and diseased plants, and changing light conditions impede the precise identification and classification of plant diseases. Meanwhile, Xie et al. (2023) detected sooty mold and other leaf diseases of *Litchi* using individual leaf images captured by smartphones in field conditions combined with an improved fully convolutional one-stage object detection (FCOS) network called FCOS for Litch (FCOS-FL) [26]. They utilized G-GhostNet-3.2 as the backbone network extraction feature to improve the FCOS that resulted in a 95.7% detection of sooty mold.

The aforementioned research methodologies produced high detection accuracies; however, they may be complicated and expensive for small farmers. Studies [19,23,24] detected different diseases with high accuracies but utilized expensive SLR, multispectral, and hyperspectral cameras that are 7×, 85×, and 220×, respectively, more expensive than the home surveillance camera. On the other hand, research [20,25,26] used semi-affordable digital cameras and smartphones to collect images; however, they lacked reliability to provide direct and simple detection methodologies and still cost 2× to 5× more than the proposed sensor camera. In addition, only the drone-attached expensive cameras provided the canopy detection of diseases and most mid-range price sensors captured individual or close-up leaf images. Thus, to build these gaps and scale down the complexity of the system as well as balance the cost with accuracy to provide a convenient, affordable, simple, and new alternative on advanced disease monitoring systems in field environment at the canopy level, this research proposes detecting sooty mold infections on the citrus canopy at night using established single-stage detectors and low-cost security surveillance cameras. Appendix A summarizes the related works cited and our proposed system for comparison.

## 3. Materials and Methods

**Figure 2:**
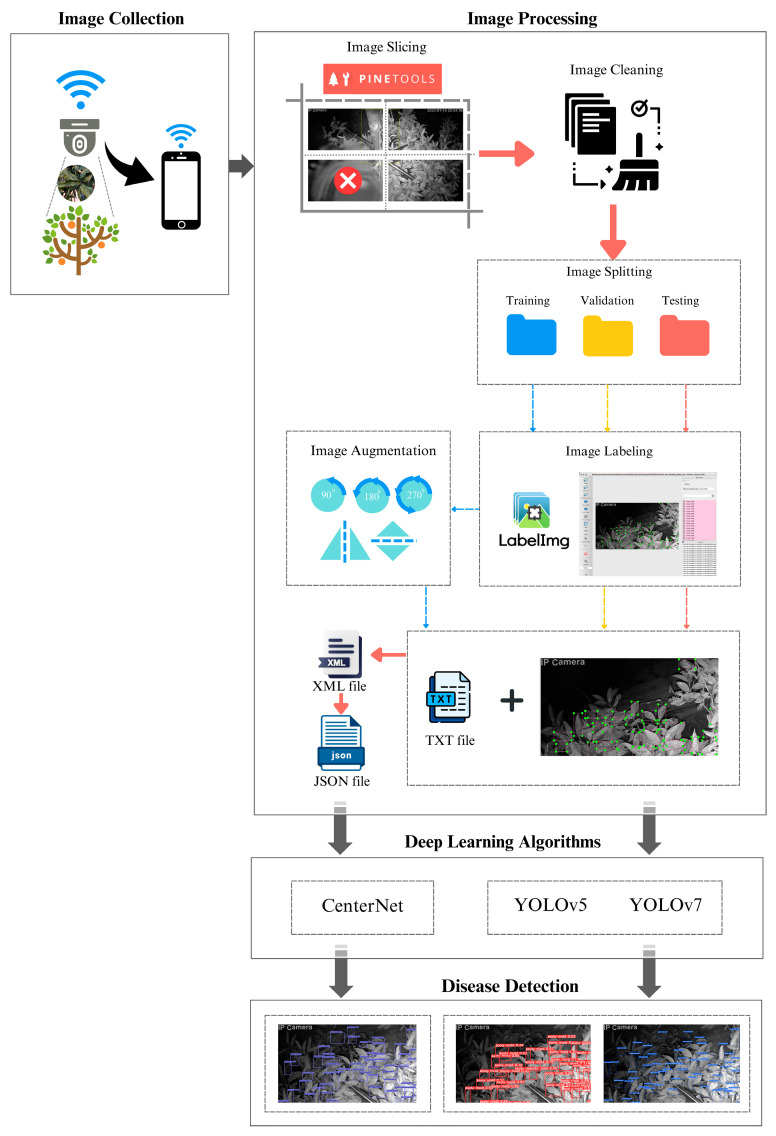
The conceptual framework for detecting sooty mold in citrus canopy using DL algorithms.

This study used Satsuma mandarin (*Citrus unshiu* Marc.) planted in Tsukuba, Ibaraki, and a home surveillance camera that was installed on a pole placed close to the trunk of the tree, high enough to capture the whole tree canopy. The Wi-Fi function was also set up to collect the data remotely. The collected images were prepared following a series of procedures that included image slicing, cleaning, splitting, augmentation, and format conversion before they proceeded to train with the three DL models (Figure 2).

### 3.1. Image Collection

**Figure 3:**
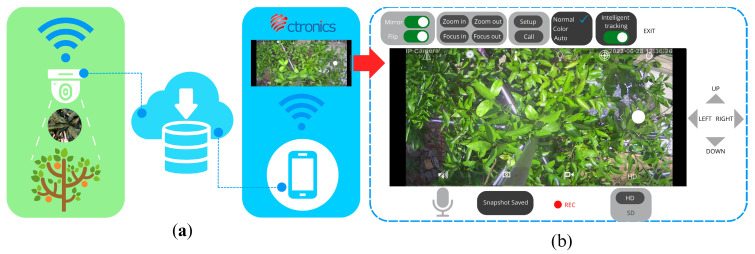
(**a**) Collection of images remotely through the CTRONICS mobile application; (**b**) CTRONICS mobile application interface for data collection.

**Figure 4:**
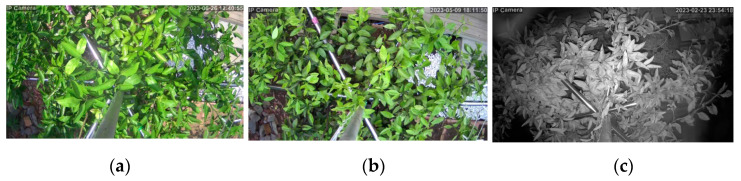
Comparison of images captured by the CTRONICS camera under different light conditions: (**a**) sunny, (**b**) cloudy, and (**c**) night.

All images were collected using a low-cost 4 MP surveillance home security camera (CTIPC-530C, Shenzhen C-TRONICS CO., LTD., Shenzhen, China) with 2560 ×1920 pixels resolution, 25 M HD night vision capability, 2 pcs IR LEDs, advanced image digital noise-reduction, Wi-Fi connectivity, and could rotate 355° horizontally and 90° vertically. To eliminate the inconvenience of traditional monitoring and data gathering in the field under changing weather conditions, IoT technology was used through the CTRONICS mobile application to remotely capture images at different angles, anytime and anywhere, provided there was a Wi-Fi connection (Figure 3). In addition, images were captured at night to minimize the effects of varying light conditions during the day on fluctuating weather conditions, causing unnecessary shadows and bright reflections on the leaves that affect the appearance of sooty mold infections. Figure 4 shows the conditions during the day and night: (a) too much light reflected on leaf surfaces and created unfavorable shadows during sunny days, (b) suitable light only on cloudy weather, and (c) images collected at night using the night vision feature of the camera produced grayscale images. Twelve images were gathered every other night (one-night interval) from 19:00 to 24:00 for six months, from October 2022 to March 2023. Each captured image had a resolution of 2560 × 1440 pixels. A total of 1080 night images were collected for the citrus canopy dataset. Unsuitable images were eliminated, and 1000 images remained and were selected to create the initial dataset that was prepared with several image processing systems.

### 3.2. Image Processing

#### 3.2.1. Image Slicing and Cleaning

The CTRONICS surveillance camera produced wide images covering the citrus canopy. These images were utilized to increase the number of datasets by slicing the images into quadrants. All 1000 images were sliced into four using Pine Tools, a free online tool that can slice single or bulk images horizontally, vertically, or both. After slicing, the resolution of each image became 1280 × 720 pixels. Moreover, the total number of images was expanded to 4000. These images underwent another round of dataset cleaning through a random selection of 1000 images, subsequently excluding poor and unsuitable images; these were blurred images due to water droplets and moisture (fog) accumulation on the camera lens.

#### 3.2.2. Image Splitting and Labeling

The images were divided into training, validation, and testing sets, where 70% (700 images) were allocated for training, 20% (200 images) for validation, and 10% (100 images) for testing. Afterward, they all underwent labeling using LabelImg, a free and open-source tool for image annotation written in Python that uses QT for the graphical interface. The black spots and build-up on the surface of the leaves that showed sooty mold infections were labeled. To provide labeling consistency, only leaves with visible and distinct symptoms were manually labeled using a bounding box to safeguard the neural network detection performance. Each image, with its own label, generated a corresponding .txt file. These files contained information about the object class and the coordinates of the bounding box, representing the upper left and lower right corners of each identified sooty mold-infected leaf.

#### 3.2.3. Image Augmentation

**Figure 5:**
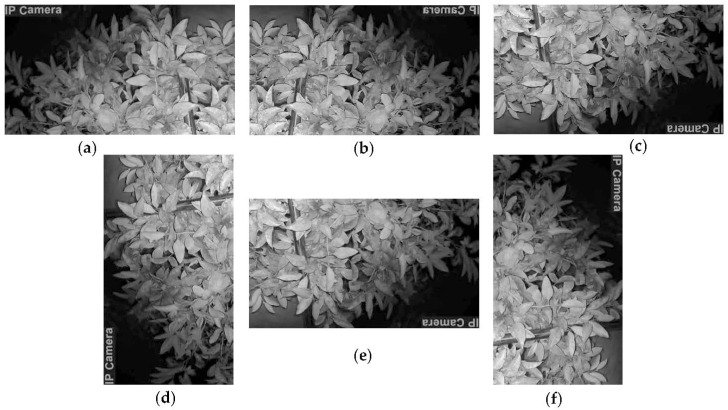
Image augmentation: (**a**) original image, (**b**) image flipped horizontally, (**c**) image flipped vertically, (**d**) image rotated 90°, (**e**) image rotated 180°, and (**f**) image rotated 270°.

A sufficient number of training images is recommended for the DL algorithms to learn better; thus, to increase the number of labeled datasets, the 700 training images were augmented by rotating to 90, 180, and 270 degrees counterclockwise and flipping horizontally and vertically (Figure 5). This 5-augmentation procedure produced 3500 additional images with corresponding .txt files that expanded the training dataset to 4200 images (Table 1). These training images, together with the unaugmented validation and testing images, were used in YOLOv5m and YOLOv7 (Table 2). Alternatively, the .txt file data of all these images were converted to XML files and then to JSON format for CenterNet training, following the same data distribution.

### 3.3. Quantitative Analysis

The three DL algorithms used in this study were YOLOv5, YOLOv7, and CenterNet. The quantitative analysis of the models was performed through the standard evaluation metrics for object detection that includes the models’ performance based on precision, recall, mean average precision (mAP), and intersection over union (IoU).

#### 3.3.1. Training Process

##### YOLOv5 and YOLOv7

**Figure 6:**
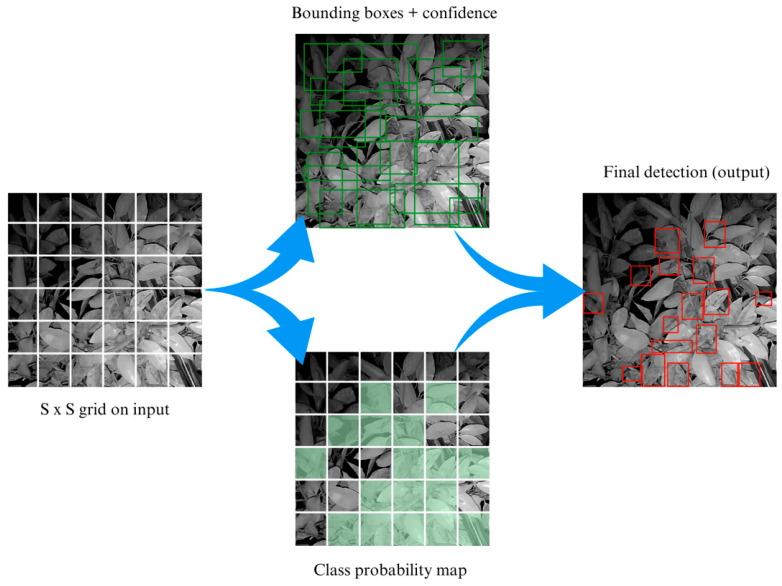
Sooty mold detection process on the YOLO algorithm.

You Only Look Once is a well-known and widely used algorithm for image detection [27]. YOLO is known for its fast computation speed and simple architecture that directly outputs the position and category of bounding boxes in the neural network (Figure 6). YOLO detects sooty mold infections by dividing the image into S × S grids. When the center of the sooty mold infection falls within a particular grid cell, the model predicts multiple bounding boxes and confidence scores for each box. However, YOLO predicts several bounding boxes per grid, thus non-maximum suppression (NMS) is applied to identify and eliminate redundant bounding boxes to achieve accurate final detection of sooty mold infections.

YOLOv5 and YOLOv7 are two recent versions that were released in 2020 and 2022, respectively. The two models have improved architecture compared to their predecessors. YOLOv5 was created using PyTorch instead of the DarkNet framework and utilized a modified CSPDarknet53 backbone [28]. YOLOv5 integrates the AutoAnchor algorithm to examine and adjust anchor boxes to ensure their compatibility with the dataset and training parameters. In addition, YOLOv5 employs a k-means function to dataset labels, generating initial conditions for a genetic evolution algorithm that refines anchor boxes over 1000 generations using complete intersection over union (CIoU) loss and the best possible recall as the fitness function [29]. YOLOv5 has five available versions: YOLOv5n (nano), YOLOv5s (small), YOLOv5m (medium), YOLOv5l (large), and YOLOv5x (extra-large). YOLOv5n is a lightweight and fast variant that emphasizes speed over performance; subsequently, as the model size increases, it progressively prioritizes high performance over speed.

Alternatively, YOLOv7 achieves better performance by utilizing an extended efficient layer aggregation network (E-ELAN) that enables effective control over the shortest longest gradient path to facilitate efficient learning and convergence of DL models even though it solely relies on the MS COCO dataset without utilizing pretrained backbones. This version also introduces several architectural modifications and a range of bag-of-freebies that enhance accuracy without impacting inference speed, albeit increasing training time. Some of these bag-of-freebies consist of planned reparametrized convolution, coarse label assignment for the auxiliary head and fine label assignment for the leading head, conv-bn-activation with batch normalization, implicit knowledge inspired by YOLOR, and the use of the exponential moving average as the final inference model [29].

##### CenterNet

CenterNet is another reliable object detection model known for anchor-free object detection that was demonstrated by Xia et al. to be suitable for detecting plant diseases in natural environments [30]. CenterNet’s algorithm differs from traditional bounding box-based approaches. CenterNet adopts a key point prediction-based detection method, where the final prediction box for plant diseases is obtained by predicting key points rather than bounding boxes [31]. In addition, instead of treating objects as pairs of key points, CenterNet detects each object as a triplet of key points, resulting in improved precision and recall. It also utilizes center pooling and cascade corner pooling to enrich the information gathered from the top-left and bottom-right corners of the object, providing more discernible details in the central regions [32]. Ultimately, identifying the final bounding boxes was accomplished by utilizing the detected center key points.

**Figure 7:**
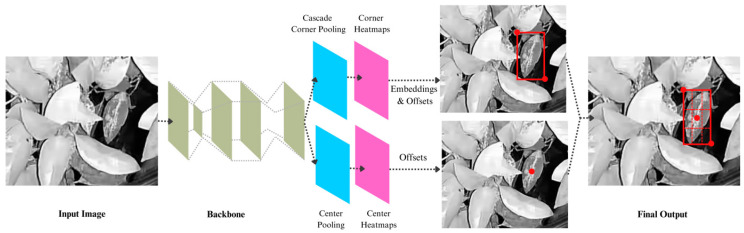
CenterNet architecture and working principles in sooty mold detection. It predicts sooty mold infections as triplet key points (red square represents the top-left and bottom-right corners; red dot represents the center key point) to produce the final detection.

CenterNet exhibits faster detection speed compared to other one-stage and two-stage detection models [33]. This is attributed to its small number of network parameters, which requires fewer computations. Figure 7 summarizes the architecture and working principles of CenterNet model disease detection.

#### 3.3.2. Performance Metrics

To evaluate the effectiveness of the model in detecting sooty mold of citrus at night at the canopy level, standard evaluation metrics for object detection, such as intersection over union (IoU), and average precision (AP), were employed. These metrics are widely used to measure model accuracy and performance.

Intersection over union (IoU) is a commonly used metric for evaluating localization accuracy and measuring localization errors in object detection models. IoU involves determining the overlap between the predicted sooty mold infections and ground-truth bounding boxes by calculating the intersection area. Through IoU, the total occurrences of true positives (TP), false positives (FP), and false negatives (FN) were defined. In this study, TP is when the sooty mold infections were detected as sooty mold, while FP is when another object was detected as sooty mold. Lastly, FN is when sooty mold infections were not detected at all.

Precision (P) measures model accuracy in detecting sooty mold by determining the proportion of TPs relative to the total number of predictions made by the model. Precision can be calculated using the equation provided below.

(1) $$\mathrm{Precision}=\frac{\mathrm{TP}}{\mathrm{TP}+\mathrm{FP}}$$

where true positives (TPs) are the correct detection and false positives (FPs) are the incorrect detection of sooty mold infections on the citrus canopy.

Meanwhile, recall assesses the model’s ability to accurately identify sooty mold among all the positive targets, including FN detections or those that were initially missed and undetected. Recall can be determined by the ratio between the total predictions of sooty molds made by the model and the total number of existing labels associated with sooty mold infections.

(2) $$\mathrm{Recall}=\frac{\mathrm{TP}}{\mathrm{TP}+\mathrm{FN}}$$

Recall and precision exhibit a tradeoff, which is visualized as a curve by adjusting the classification threshold of sooty mold infections on citrus canopy. The area under this precision-recall curve represents the average precision for sooty mold infections in the model. Taking the average of these values across all classes gives the mean average precision (mAP), which can be calculated using the following expression:

(3) $$\mathrm{mAP}=\frac{1}{C}\sum_{k=1}^{T}P(k)∆R(k)$$

where C is the total class number, T is the IoU threshold number of sooty mold infections, k is the IoU threshold of sooty mold infections, P(k) is the precision, and R(k) is the recall.

### 3.4. Qualitative Analysis

Qualitative analysis of the best-performing model was also conducted on enhanced images (50% and 80% brightness) to test the robustness of the model in detecting sooty mold infections at night at varying levels of brightness.

## 4. Results

### 4.1. Quantitative Analysis

#### 4.1.1. Training

This study utilized a dataset comprising 4500 images, with 4200 images allocated for training, 200 images for validation, and 100 images for testing purposes. These images were processed using three object detection models, namely, YOLOv5m, YOLOv7, and CenterNet, following the configuration described in Table 3.

Table 4 summarizes the results of the training process that shows the performance of the DL algorithms on validation results at 50% IoU. Considering the three parameter sets, precision, recall, and mAP, YOLOv7 shows a slight advantage in overall performance compared to the other two models. YOLOv7 accounted for a 69.8% recall value with 74.4% mAP compared to YOLOv5m with a 66.7% recall value and 72% mAP. In contrast, CenterNet generated the lowest recall and mAP values of 52.7% and 70.3%, respectively.

**Figure 8:**
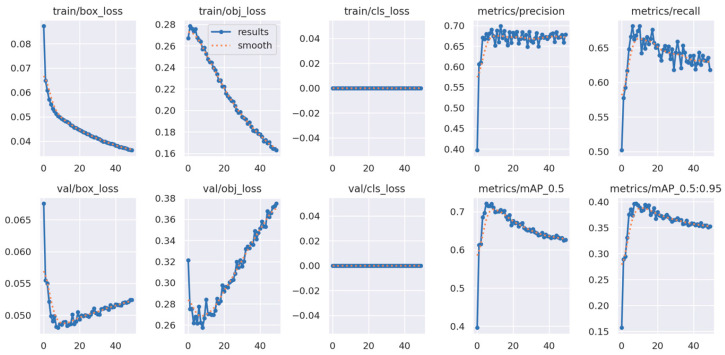
Training and validation results from YOLOv5m.

**Figure 9:**
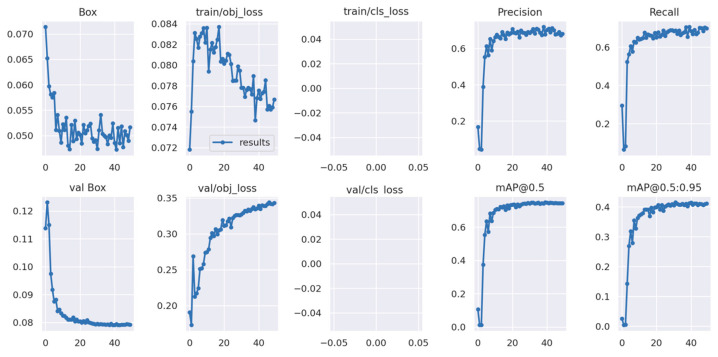
Training and validation results from YOLOv7.

Figure 8 and Figure 9 show the training curves for YOLOv5m and YOLOv7, respectively. Both exhibited a favorable performance in detecting sooty mold on citrus canopy, as evident in the decline in training losses and improvement in mAP over time, demonstrating the models’ development on learning to identify and detect sooty molds accurately. However, validation losses of both models progressed, which might be attributed to the limited diversity of the image dataset used during the training.

In addition to the abovementioned parameters that were used to determine the best algorithm among YOLOv5m, YOLOv7, and CenterNet for detecting sooty mold in citrus at night, the training time and size of each model were also considered in deciding the appropriate model for specific training situations (Table 5). YOLOv5m and YOLOv7 trained at similar rates of 6.3 and 7.1 h, respectively, compared to the longer training hours of CenterNet, which reached up to 67 h to complete. Moreover, the size of each model also differed; YOLOv5m was the lightest at 40.2 MB, and CenterNet was the heaviest at 83.9 MB. These additional parameters together with precision, recall, and mAP provide valuable information that can help us choose the most suitable model for detecting sooty mold infections at night on citrus canopies in orchard environments.

#### 4.1.2. Model Testing

To evaluate the performance of the three trained models in detecting sooty mold in the citrus tree canopy at night, 100 original images were used for testing using the best-trained weight from each model. The results show that YOLOv7 was the most accurate in detecting sooty molds compared to YOLOv5m and CenterNet. YOLOv7 achieved 75.6% mAP, while YOLOv5m and CenterNet scored slightly lower, with 67.9% and 72.3% mAP, respectively (Table 6).

#### 4.1.3. Testing on Preprocessed (Unsliced) and Day-Captured Images

To further evaluate the performance and accuracy of the trained algorithms, the best-trained weight of each model was tested on 10 randomly selected preprocessed (unsliced) night images. The test shows a similar detection trend with the sliced images, and YOLOv7 leads the detection at 60.3% mAp (Table 7). Interestingly, CenterNet had an underwhelming performance at 27.5% mAP.

Moreover, to push the boundaries and determine the possible limitations of the models, day-captured images were tested to determine the accuracy and competence of the models in detecting sooty mold infections. The results demonstrated unfavorable outcomes for detecting sooty molds for all the DL models for both sliced and unsliced images. All models had low mAPs compared to their night counterparts. However, YOLOv7 performed better than the other two algorithms at 42.5% and 26.7% mAP on sliced and unsliced images, respectively (Table 8).

These results also demonstrated that CenterNet had a weak performance on unsliced images both for night- and day-captured images; however, it performed similarly to YOLOv7 and better than YOLOv5m on sliced images. The low detection of the models, especially on day-captured images, is not surprising given that the images had a different environment and were totally different from the images used in the training.

**Figure 10:**
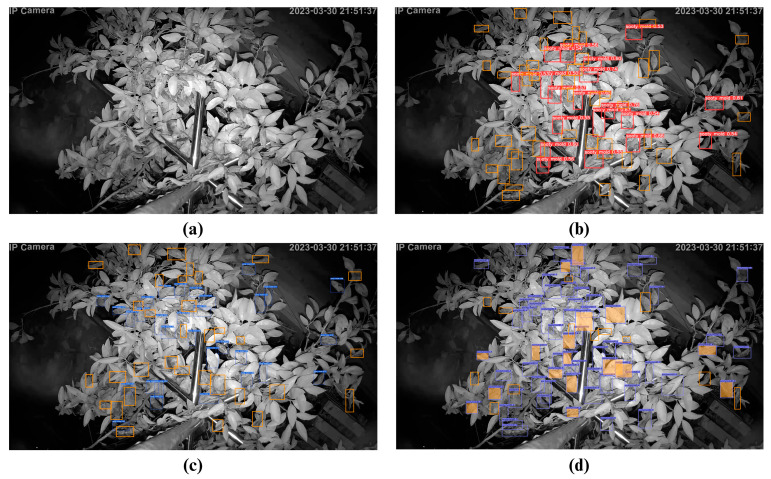
Test examples on the detection of sooty molds using preprocessed (unsliced) images from (**a**) Original image, (**b**) YOLOv5m, (**c**) YOLOv7, and (**d**) CenterNet. (The orange square in the figure refers to FN or undetected sooty mold infections, and the orange fill indicates false positive (FP) detection.)

Figure 10 and Table 9 showcase sample results of the models in detecting sooty molds on citrus using 10 preprocessed (unsliced) night images. From 64 total sooty mold infections, CenterNet detected 50, the highest detection among the three models; however, it also acquired 17 false positive (FP) detections compared to none with the other two models. Weighing the correct and false detections, YOLOv7 still performed better than the two other models, with 31 detections and 33 undetected sooty mold infections.

**Figure 11:**
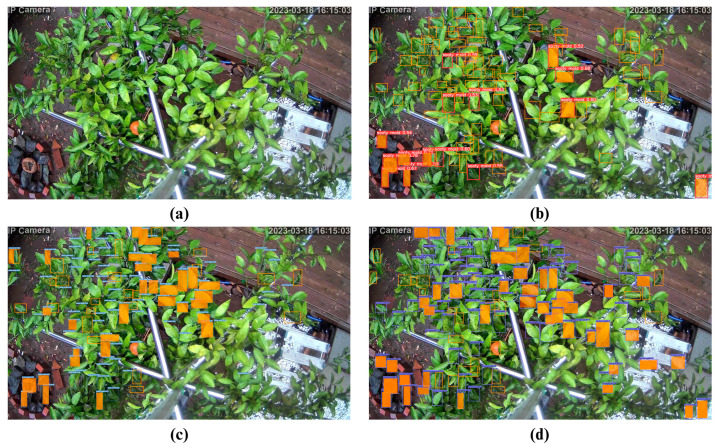
Test examples on the detection of sooty molds using unsliced day-captured images from (**a**) original image, (**b**) YOLOv5m, (**c**) YOLOv7, and (**d**) CenterNet. (The orange square in the figure refers to FN or undetected sooty mold infections, and the orange fill indicates false positive (FP) detection.)

**Figure 12:**
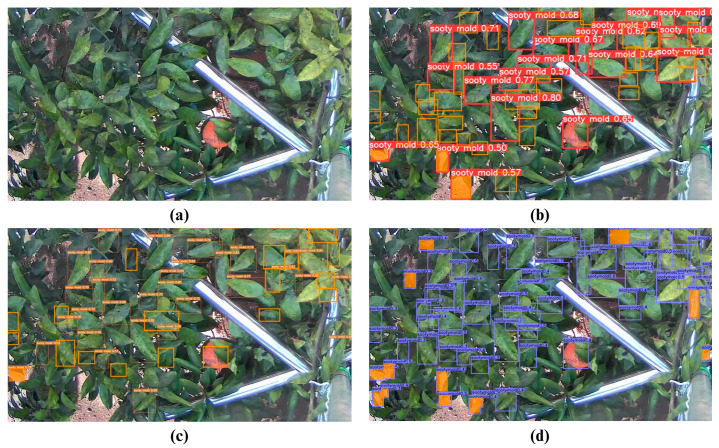
Test examples on the detection of sooty molds using sliced day-captured images from (**a**) original image, (**b**) YOLOv5, (**c**) YOLOv7, and (**d**) CenterNet. (The orange square in the figure refers to FN or undetected sooty mold infections, and the orange fill indicates false positive (FP) detection.)

For the day-captured images, testing results on the full/unsliced version resulted in 58 detections for YOLOv7, 53 for CenterNet, and only 5 for YOLOv5m, out of the total of 79 sooty mold infections (Figure 11 and Table 10). It is also worth noting that YOLOv5m had the highest undetected infections at 74, while CenterNet topped the misdetection result at 44. In contrast, on day-captured sliced images, CenterNet detected all 51 infections but incurred 13 false detections (Figure 12 and Table 11). YOLOv5m and YOLOv7 had only 3 and 1 false detections, respectively. These results show that using a night image-trained model to detect sooty mold infections in day-captured images faces difficulty, causing low accuracy in detection, including high false detection.

### 4.2. Qualitative Analysis

**Figure 13:**
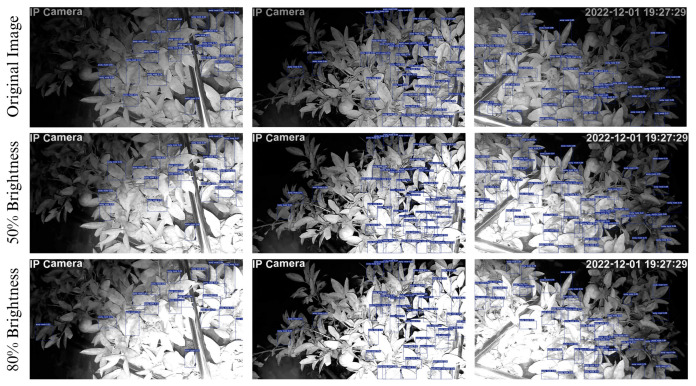
Visual Comparison of the Performance of YOLOv7 on original and enhanced images (50% and 80% brightness).

To further assess the performance and robustness of the best sooty-mold detection model, YOLOv7, a qualitative analysis was performed by subjecting the model to images under varying brightness levels. To conduct the analysis, a set of test images was processed, systematically altering their brightness levels. The original images were enhanced to 50% and 80% brightness, and consequently tested on the best weight of YOLOv7. Figure 13 shows the original images in the first row and the second and third row represent 50% and 80% brightness, respectively. The visual representation demonstrated the outstanding performance of YOLOv7 on how well it responded to varying light intensities, highlighting the models’ ability to detect sooty mold accurately at night regardless of the lighting conditions. Comparing the original image detection to 50% and 80% brightness, the majority of the infections were still detected by the model despite the increased image brightness.

## 5. Discussion

### 5.1. Performance Analysis of the Three DL Algorithms

Conventional disease monitoring and detection methods are laborious, prone to inaccuracies, and necessitate on-site visual assessment by experienced experts, which can be costly and inconvenient. Thus, this study used available IoT and DL technologies to detect sooty mold infection on citrus canopy to provide affordability, accuracy, and convenience. Images were captured remotely using IoT technology through the CTRONICS mobile application. In addition, images captured at night were used to build the training dataset, as the field environment is often subjected to varying light conditions, especially during the day, which affects the quality of images captured by the camera and the appearance of sooty mold infections on the citrus canopy. This is a crucial concern in object detection, as the images must be learned and recognized properly by the computer algorithm. This study used images captured at night using an affordable home surveillance camera with night vision capability. The night vision component of the camera automatically transformed the images into black and white, highlighting the occurrence of sooty molds on leaf surfaces. It also clearly separated the leaves from the background and minimized shadows that are dominant in images captured during the day. This makes image collection and processing simple and straightforward, as it eliminates additional procedures such as image enhancement in dataset preparation.

The night-captured image dataset was trained on three established object detection models: YOLOv5m, YOLOv7, and CenterNet. Based on the parameter set, the YOLOv7 model had the best overall performance in terms of training detection accuracy at 74.4% and model testing at 75.6%. Likewise, it had a fast-training time of 7.1 h, similar to YOLOv5m at 6.3 h, and was remarkably quicker than the 67 h training time of CenterNet. However, YOLOv7 is not the lightest model among the groups and is situated in the middle of YOLOv5m (40.2 MB) and CenterNet (83.9 MB) at 71.3 MB. The size of one-stage object detection models, in general, was manageable compared to the large model size of two-stage detection models and can be considered for an orchard real-time disease monitoring and detection system. The reason behind this is that a lighter model usually has fewer parameters that require less computational resources and training time compared to larger models. YOLOv7 has a less complex architecture and needs less data for training. Thus, YOLOv7 is highly recommended in orchard real-time disease monitoring and detection for faster, simpler, and more practical applications.

However, the results in Figure 8 and Figure 9 showed some unstable plots and a lack of decaying in validation losses that were encountered due to the limited data diversity. When training a deep learning model, having a diverse and comprehensive dataset is important for the algorithm to generalize well across different scenarios. In the case of this study, the limited dataset might restrict the model’s ability to learn complex patterns effectively during training, leading to unstable validation losses.

Nevertheless, it is worth noting that the model can still perform well on unseen data during testing (Table 6). This indicates that the model had learned valuable features and was capable of generalizing new, previously unseen instances. Even so, to address this challenge and further improve the models’ performance, there are several strategies that will be considered further, such as adding a greater number of trees and selecting different locations to create a diversified dataset.

### 5.2. Performance Analysis on the Detection Using Preprocessed and Day-Captured Images

The performance of the three DL models using preprocessed (unsliced) night images was more reliable and consistent compared to the day-captured images. This result was expected, as the training dataset was derived from this version, and they had the same lighting conditions that heavily favored similar detection, as image characteristics such as contrast, color, and texture were the same [28]. This is a promising result for building real-time orchard disease monitoring and detection, as the images or videos captured by the surveillance camera can directly detect sooty mold infection without slicing the image.

In contrast, poor detection of sooty mold on citrus canopy was observed using day-captured images on both sliced and unsliced versions. The results were expected, as the dataset used in training consisted of night-captured images that have a completely different color and lighting condition compared to the day-captured images. The low detection of sooty mold infections showed the limitation of the models to build a training dataset based on night-captured images that can detect sooty mold during the day. In terms of the model performance, YOLOv7 consistently topped the detection accuracy across the three types of image datasets, outperforming YOLOv5m and CenterNet, and had more reliable testing results than the erratic detection of CenterNet and low detection of YOLOv5m. CenterNet had a weak detection accuracy on unsliced images regardless of light condition, which was attributed to the limitation of the model in detecting small objects. Unsliced images were the combination of 4 sliced images; hence, sooty mold infections appeared smaller, uniform, and more complex on unsliced images, which caused difficulty in extracting reliable sooty mold features affecting CenterNet’s performance [34].

YOLOv5m had higher precision results on testing using preprocessed and day-captured images compared to YOLOv7. However, the high precision of YOLOv5m caused fewer detections and low true positive results on testing due to the high sensitivity of the model to detect sooty mold infections on the images. The YOLOv5m model tuned for high precision to minimize false positives ensured that most of the predicted positive instances were correct. However, aiming for high precision can come at the cost of missing some true, positive sooty mold infections, resulting in low recall. In other words, the YOLOv5m model was very cautious in making positive sooty mold predictions and did not detect sooty mold infections if it was not very confident about the presence of sooty mold.

Moreover, the testing results of the models showed some misdetections, which can be attributed to labeling inconsistencies, as not all sooty mold infections were clear due to moisture fogging and water droplets covering some parts of the lens, in addition to limitations on the camera resolution, camera angle, distance from the canopy, and other pest and disease damage.

## 6. Conclusions

In citrus disease management, sooty mold is one of the significant causes of the decrease in the production and quality of fruit. To support fruit growers in controlling the disease at an early stage, an onsite, low-cost, and real-time data collection platform is required. However, image data transmission and dataset quality must be stable and protected. To overcome these challenges on convenience, quality, and cost, this research aimed to bring a solution using an affordable surveillance camera combined with one-stage detection algorithms to remotely monitor and detect sooty mold on the citrus canopy at night in the field environment. The proposed methodology used a night-time image dataset, reducing the need for extensive image preprocessing. The images that were collected at night minimized the effects of varying light conditions, averting the need for expensive cameras and complicated algorithms to detect sooty mold infections. The images were also collected remotely using IoT technology to eliminate the need for an on-site data collection. The study found that a low-cost camera sensor can provide reliable detection of citrus sooty mold infections using simple DL models. Among the utilized DL algorithms, YOLOv7 performed the best accuracy at 74.4% on detecting citrus sooty mold at night compared to YOLOv5m and CenterNet. Moreover, YOLOv7 consistently performed better on testing with preprocessed night and day images. The study plans to further improve the accuracy of the models in sooty mold detection using multilocation datasets and develop a holistic disease management system that aims to monitor, detect, count, and control disease incidence. In further research, the system will incorporate a DL counting mechanism to calculate the canopy area with the highest number of infections for implementing a spot spraying system that can apply the right amount of control on sooty mold infections at the right time.

## Figures and Tables

**Table 1:** Total number of image datasets before and after augmentation.

Image	Training	Validation	Testing	Total
Original Image	700	200	100	1000
Augmented Image	3500	0	0	3500
Total	4200	200	100	4500

**Table 2:** Image data allocation for DL algorithms.

Model	Training	Validation	Testing
YOLOv5m	4200	200	100
YOLOv7	4200	200	100
CenterNet	4200	200	100

**Table 3:** Training configuration for three object detection models.

Model	Input Size	Batch Size	Epoch
YOLOv5m	640 × 640	16	50
YOLOv7	640 × 640	16	50
CenterNet	512 × 512	-	150

**Table 4:** DL algorithm training performance in detecting sooty molds in citrus canopy.

Model	Precision (%)	Recall (%)	mAP (%)
YOLOv5m	69.0	66.7	72.0
YOLOv7	68.1	69.8	74.4
CenterNet	-	52.7	70.3

**Table 5:** Training time and size of the object detection models.

Model	Training Time	Model Size
YOLOv5m	6.3 h	40.2 MB
YOLOv7	7.1 h	71.3 MB
CenterNet	67 h	83.9 MB

**Table 6:** DL algorithm testing performance in detecting sooty molds in citrus canopy.

Model	Precision (%)	Recall (%)	mAP (%)
YOLOv5m	78.1	57.1	67.9
YOLOv7	66.9	72.5	75.6
CenterNet	-	54.4	72.3

**Table 7:** Testing performance on preprocessed (unsliced) night images.

Model	Night-Unsliced Image		
	Precision (%)	Recall (%)	mAP (%)
YOLOv5m	74.1	24	47.6
YOLOv7	59.7	63.3	60.3
CenterNet	-	25.3	27.5

**Table 8:** Testing performance using day-captured images.

Model	Day-Sliced Image			Day-Unsliced Image		
	Precision(%)	Recall (%)	mAP (%)	Precision (%)	Recall (%)	mAP (%)
YOLOv5m	53.1	19.7	34.1	35.1	5.4	19.1
YOLOv7	45.5	51.0	42.5	30.6	48.0	26.7
CenterNet	-	44.2	41.8	-	17.8	13.8

**Table 9:** Summary of the detection results of the three models on the sample preprocessed night image.

Model	Total of Sooty Mold Labels	True Positive	False Negative	False Positive
YOLOv5m	64	19	45	0
YOLOv7	64	31	33	0
CenterNet	64	50	14	17

**Table 10:** Summary of the detection results of the three models on the sample unsliced day-captured image.

Model	Total of Sooty Mold Labels	True Positive	False Negative	False Positive
YOLOv5m	79	5	74	10
YOLOv7	79	58	21	34
CenterNet	79	53	26	44

**Table 11:** Summary of the detection results of the three models on the sample sliced day-captured image.

Model	Total of Sooty Mold Labels	True Positive	False Negative	False Positive
YOLOv5m	51	16	35	3
YOLOv7	51	33	18	1
CenterNet	51	51	0	13

## Data Availability

The dataset that was generated and used in this study is available on request from the corresponding author, but restrictions apply to data reproducibility and commercially confidential details.

## Appendix A

**Table A1:** Summary of the related works cited and our proposed system for comparison.

Article	Crop	Features	Algorithms/ Models	Accuracy	Sensor	Captured Image; Environment
Uğuz et al., 2023 [18]	Citrus	Disease detection and physical disorders caused by *Alternaria alternata* and thrip diseases classification for citrus fruit images using convolutional neural network.	Faster RCNN with ResNetX101-FPN; Mask R-CNN with ResNetX101-FPN; SSD300 with VGG16; YOLOv5 with CSPDarknet	74.3% 98.8% 93.2% 99%	Not mentioned	Individual fruit; Controlled environment
Soeb et al., 2023 [19]	Tea	Used an improved YOLOv7 object detection model, YOLO-T, for tea leaf diseases detection in natural environment.	YOLOv7	97.3%	Canon EOS 80D SLR camera	Close-up leaf image; Field environment
Dananjayan et al., 2022 [20]	Citrus	Developed a new dataset of infected citrus leaves with precise annotations and multiple classes called CCL’20 on single leaf images to detect anthracnose, melanose and bacterial brown spot.	CenterNet2 detector with ResNeXt-101, DCN/Res2Net-101, DCN/Res2Net-101 DCN-BiFPN Others…	89%, 90%, 91%	Point and shoot camera	Individual leaf; Controlled environment
Syed Ab Rahman et al., 2022 [21]	Citrus	Employed a two-step, deep CNN to detect citrus black spot, citrus bacterial canker, and Huanglongbin.	Faster R-CNN	94.3%	Not mentioned	Individual leaf; Controlled environment
Abdulridha et al., 2019 [23]	Citrus	UAV-based remote sensing technique to detect citrus canker.	Neural Network Radial Basis Function (RBF); K-Nearest Neighbor (KNN)	100% 96%	Resonon Pika L 2.4 hyperspectral camera mounted on a UAV (DJI Matrice 600, Pro Hexacopter)	Canopy; Field environment
DadrasJavan et al., 2019 [24]	Citrus	Detected citrus greening on low-altitude multispectral images using multispectral camera attached to UAV.	SVM	81.7%	Micasense RedEdge multispectral camera attached to UAV	Canopy; Field environment
Khanramaki et al., 2021 [25]	Citrus	Proposed ensemble of deep learning models that involved three levels of diversity including classifier level, feature level, and data level diversity to detect leafminer, pulvinaria, and sooty mold.	AlexNet, VGG16, ResNet 50, Inception-ResNet-v2, Proposed ensemble	91.3% 89.3% 96.1% 92.8% 99%	Sony DSC-W170, Samsung Galaxy J7, Samsung Galaxy Grand Prime, and X6 Apple iPhone	Individual leaf; Field environment
Xie et al., 2023 [26]	Litchi	Used an improved FCOS network called FCOS for Litch (FCOS-FL) with G-GhostNet-3.2 as the backbone network to detect to detect sooty mold and other diseases.	FCOS-FL	95.7%	iPhone 12 and Xiaomi 6	Individual leaf: Field environment
Proposed system	Citrus	Sooty mold detection on citrus canopy at night using affordable surveillance camera and one-stage object detection models.	YOLOv5m YOLOv7 CenterNet	72% 74.4% 70.3%	CTRONICS home surveillance camera	Canopy; Field environment
